# Treatment of Alzheimer's disease with framework nucleic acids

**DOI:** 10.1111/cpr.12787

**Published:** 2020-03-12

**Authors:** Xiaoru Shao, Weitong Cui, Xueping Xie, Wenjuan Ma, Yuxi Zhan, Yunfeng Lin

**Affiliations:** ^1^ State Key Laboratory of Oral Diseases National Clinical Research Center for Oral Diseases West China Hospital of Stomatology Sichuan University Chengdu China; ^2^ Affiliated Hospital of Jining Medical University Jining Medical University Jining China

**Keywords:** Alzheimer's disease, blood‐ brain barrier, framework nucleic acids, nanoparticle

## Abstract

**Objectives:**

To provide a new research direction for nerve regeneration and strategy for Alzheimer's disease treatment, tetrahedral DNA nanostructures (TDNs)—novel tetrahedral framework nucleic acid molecule nanoparticles (tFNA) that can inhibit the apoptosis of nerve cells are employed in the experiment.

**Materials and methods:**

To verify the successful preparation of TDNs, the morphology of TDNs was observed by atomic force microscopy (AFM) and transmission electron microscopy (TEM). The expression of apoptosis‐related genes and proteins was investigated by confocal microscope, flow cytometry, PCR and Western blot to detect the impact of TDNs on the Alzheimer's model. And finally, Morris water maze experiment was used to test behavioural changes and Nissl stain was detected to observe the morphology and quantity of neurons in the hippocampus. Immunofluorescence stain was used to observe the Aβ stain, and TUNEL dyeing was utilized to observe neuronal apoptosis.

**Results:**

In vitro and in vivo experiments confirm that TDNs, in a specific concentration range, have no toxic or side effects on nerve cells, can effectively inhibit apoptosis in an Alzheimer's disease cell model and effectively improve memory and learning ability in a rat model of Alzheimer's disease.

**Conclusions:**

These findings suggest that TDNs may be a promising drug for the treatment of Alzheimer's disease.

## 1 INTRODUCTION

Alzheimer's disease is one of the most common age‐related neurodegenerative diseases.^1^ Its clinical manifestations include progressive memory impairment, cognitive dysfunction, personality changes and language disorders. Studies have shown that apoptotic neuronal death is an important component of Alzheimer's disease, as neuronal apoptosis is discovered in early Alzheimer's disease stages, and a large number of apoptotic neurons in the cerebral cortex and hippocampus have also been shown in autopsy of Alzheimer's disease patients.^2^, ^3^ Alzheimer's disease pathogenesis is still unclear, but the most broadly accepted hypothesis involves the beta‐amyloid (Aβ) cascade. According to this hypothesis, the accumulation of Aβ in the brain is the main cause of induction and aggravation of Alzheimer's disease. When Aβ aggregates to form brain sediments, it causes neurodegenerative diseases, leading to clinically observed loss of patient memory and cognitive ability. Aβ aggregation can cause a series of pathological changes, which ultimately induces cytotoxicity and cell death, including mitochondria‐dependent apoptosis.^4^, ^5^ Studies have shown that some drugs for Alzheimer's disease treatment may cause cytotoxicity (eg, tacrine can cause significant hepatotoxicity); therefore, we aimed to find a new drug that can inhibit nerve cell apoptosis and thus become a safer option for the treatment of Alzheimer's disease.^6^, ^7^, ^8^

Tetrahedral DNA nanostructures (TDNs) are novel three‐dimensional framework nucleic acids (tFNA) which currently have broad application prospects in the biomedical field. The advantages of TDNs include simple synthesis, high yield, good biocompatibility and good in nuclease resistance.^9^, ^10^, ^11^, ^12^ Fortunately, according to previous literature reports, TDNs can partially pass the blood‐brain barrier (BBB),^13^ a special structure that separates the central nervous system (CNS) from the blood circulation and prevents substances in the plasma that are toxic to brain tissue from entering the brain, thereby maintaining a good living environment for nerve cells. At present, there is a lack of reports on the role of TDNs in the treatment of Alzheimer's disease.

Based on the above research, we focused on the therapeutic role of TDNs in Alzheimer's disease and performed both in vitro and in vivo experiments in Alzheimer's disease models. Our results confirm that TDNs exert an inhibitory effect on apoptosis both in a PC12‐cell Alzheimer's disease model and in the hippocampus of an Alzheimer's disease rat model. Moreover, our experimental results show that the underlying mechanism of TDN function in these Alzheimer's disease models involves the inhibition of the mitochondria‐dependent apoptotic pathway.

## 2 MATERIALS AND METHODS

### 2.1 Materials

TDNs were fabricated on the basis of a previous method.^14^, ^15^ The capillary electrophoresis technique (Qsep 100TM; Bioptic) and non‐denaturing polypropylene gel electrophoresis were used to verify successful TDN synthesis, by measuring the molecular weight of DNA single strand and TDN. Subsequently, we measured TDN particle size by dynamic light scattering (DLS).

### 2.2 Real‐time cell analysis

PC12 cells were seeded into Real‐time cell analysis (RTCA)‐specific culture plates at a concentration of 5000 cells/well and cultured in an incubator at a temperature of 37°C and a carbon dioxide concentration of 5% for 48 hours. Next, Aβ was added to PC12 cells at a concentration of 25 μmol/L for 24 hours to generate an Alzheimer's disease cell model, as previously described.^16^ After Alzheimer's disease was successfully modelled, the well plate was rinsed 3 times with 0.02 mol/L phosphate‐buffered saline (PBS), and TDN was added at a concentration of 250 nmol/L. At the corresponding time points, the corresponding values on the RTCA apparatus were used for analysis.

### 2.3 Detection of apoptosis by flow cytometry

After 24 hours of TDN or serum‐free medium treatment, cells were digested with trypsin containing no EDTA and collected in a 15 mL centrifuge tube (300 *g*, 5 minutes). The supernatant was discarded, washed with PBS and centrifuged (300 *g*, 5 minutes). The cell pellet was suspended in 400 μL of Annexin V binding solution at a concentration of approximately 10^6^ cells/mL. Then, 5 μL of Annexin V‐FITC staining solution was added to the cell suspension and gently mixed at 4°C for 15 minutes in the dark, followed by addition of 5 μL propidium iodide staining solution, gentle mixing and incubation at 4°C for 5 minutes in the dark. Subsequently, cells were transferred to a flow cytometry tube and analysed using a flow cytometer (FC500 Beckman).

### 2.4 Immunofluorescence analysis

Cells were fixed with 4% paraformaldehyde at 37°C for 20 minutes, incubated with 0.5% Triton X‐100 for 15 minutes and blocked with 5% skimmed milk powder at 37°C for 50 minutes before adding the primary antibodies against caspase‐3 (1:100; Abcam) and Bax (1:100; Abcam). After incubation at 4°C overnight, cells were incubated with a secondary antibody (1:2000, Beyotime) for 1 hour at room temperature. Finally, cells were stained with phalloidin and DAPI to observe the cell skeleton and nucleus and photographed using a fluorescence or a laser confocal microscope (N‐SIM, Nikon).

### 2.5 Alzheimer's disease rat model generation

Sprague‐Dawley rats were anesthetized with an intraperitoneal injection of 1% sodium pentobarbital at an injection rate of 40 μg/g and then fixed on a brain stereotaxic instrument. The hippocampal CA1 area was slowly and uniformly injected, bilaterally, with 10 μg (1 μL) of Aβ1‐40 or the same amount of normal saline (1 μL; sham operation). The needle was left for 5 minutes, and the wound was sutured after withdrawing the needle. Three days after the operation, rats were randomly divided into two groups: the first received a daily tail‐vein injection of 100 μL normal saline for 21 days, and the second an injection of 100 μL TDN, daily, for 21 days. The blank group does not do anything. All animal experiments were performed in accordance with NIH Laboratory Animal Care and Use Guidelines and approved by the Animal Experimental Ethics Committee of Sichuan University.

### 2.6 Western blotting

After different treatments, PC12 cell and brain tissue lysate were prepared, and protein samples were separated by SDS‐PAGE electrophoresis and transferred onto PVDF membranes. Membranes were placed in blocking solution and then incubated with corresponding primary antibody (anti‐caspase‐3 [ab13847], anti‐Bcl‐2 [ab196495] or anti‐Bax [ab32503]; Abcam). The next day, membranes were rewarmed at room temperature for 1 hour and incubated with the appropriate secondary antibody (A0208, A0216, Beyotime) for 1 hour at 37°C. Finally, membranes were incubated with exposure liquid (1705060, Bio‐Rad) and developed using enhanced chemiluminescence detection system (Bio‐Rad). The data were processed using Quantity One software.

### 2.7 Quantitative real‐time PCR

Total RNA was isolated from PC12 cells or collected brain tissues with the RNeasy Mini Kit (Qiagen). Then, cDNA was prepared by reverse polymerase chain transcription using the Qiagen One‐step RT‐PCR Kit (Qiagen). All operations were performed according to previously described methods,^17^ and the expression of caspase 3, Bcl‐2 and Bax was determined by real‐time quantitative real‐time PCR (qPCR; ABI 7300). The primer sequences for each gene are displayed in Table 1.

**Table 1:** The primers sequences of relevant genes designed for qPCR

Primer name	Direction	Primer sequence
Caspase‐3‐F	5′‐3′	CGGACCTGTGGACCTGAAAA
Caspase‐3‐R	5′‐3′	TAACCGGGTGCGGTAGAGTA
Bcl‐2‐F	5′‐3′	CTGGTGGACAACATCGCTCT
Bcl‐2‐R	5′‐3′	GCATGCTGGGGCCATATAGT
Bax‐F	5′‐3′	CACTAAAGTGCCCGAGCTGA
Bax‐R	5′‐3′	CTTCCAGATGGTGAGCGAGG
GAPDH‐F	5′‐3′	TTCCTACCCCCAATGTATCCG
GAPDH‐R	5′‐3′	CATGAGGTCCACCACCCTGTT

### 2.8 Detection of the ability to penetrate the blood‐brain barrier

We used a method of co‐culture of human micro vascular endothelial cell line bEnd.3 and PC12 cells to establish a model of blood‐brain barrier in vitro. The bEnd.3 cells were cultured in the upper chamber and cultured for seven days until a tight junction was formed between bEnd.3 cells. Then, the bEnd.3 cells were co‐cultured with a Petri dish inoculated with PC12 cells, and finally, Cy‐5‐labelled TDN was added to the upper bEnd.3 cells. After 24 hours, we detected the fluorescent TDN in PC12 cells by flow cytometry, reflecting the ability of TDN to pass the blood‐brain barrier. In addition, we injected fluorescent TDN through the tail vein and observed the distribution of TDN in rats in real time by small animal in vivo imaging.

### 2.9 Behavioural testing

For all groups, the Morris water maze test was performed after 21 days saline administration or TDN administration.^18^ The test consisted of two phases: a positioning navigation phase and a space exploration phase. In the first, rats received 5 consecutive days of training in the water maze, 4 times a day, 30 minutes each, and the time required to enter the water from four water inlet points and find the platform. The average score of the four trials in each day was used for the final statistical analysis as the final result of the day. In the space exploration phase, on the 6th day of the experiment, the platform was removed, and after entering the water from the farthest end of the platform, the rats were placed in water, the swimming trajectory of the rats within 30 seconds was recorded, and the rats were observed and stayed in the target quadrant. Time, the number of times it traversed the platform and the latency it first found on the platform.

### 2.10 Nissl staining

After the behavioural testing, rats in each group were sacrificed, and the whole brain was removed, and the cerebellum was cut off on ice. We first made paraffin sections based on the steps in the literature report.^19^ Subsequently, we performed Nissl staining on the sections. Sections were microscopically examined using upright optical microscope (CK31; Olympus), and images were captured for analysis.

### 2.11 TUNEL staining

The frozen sections were fixed with 4% paraformaldehyde for 20 minutes and incubated on ice for 2 minutes in a permeabilization solution. Subsequently, 50 μL of a freshly prepared TUNEL (TdT‐mediated dUTP Nick‐End Labelling) solution was added onto each slide. Slides were finally examined under a fluorescence or laser confocal microscope. The apoptotic rate was calculated by analysing the relevant hippocampus parts of the sample by microscopy.

### 2.12 Statistical analysis

Data analysis was performed using SPSS 16.0 statistical software. All data are expressed as mean ± standard deviation. All groups were compared using multivariate analysis of variance. One‐way analysis of variance was used to compare the effect of TDNs at different time points in the same group. *P* values <.05 were considered as statistically significant.

## 3 RESULTS

### 3.1 Successful TDN synthesis

**Figure 1:**
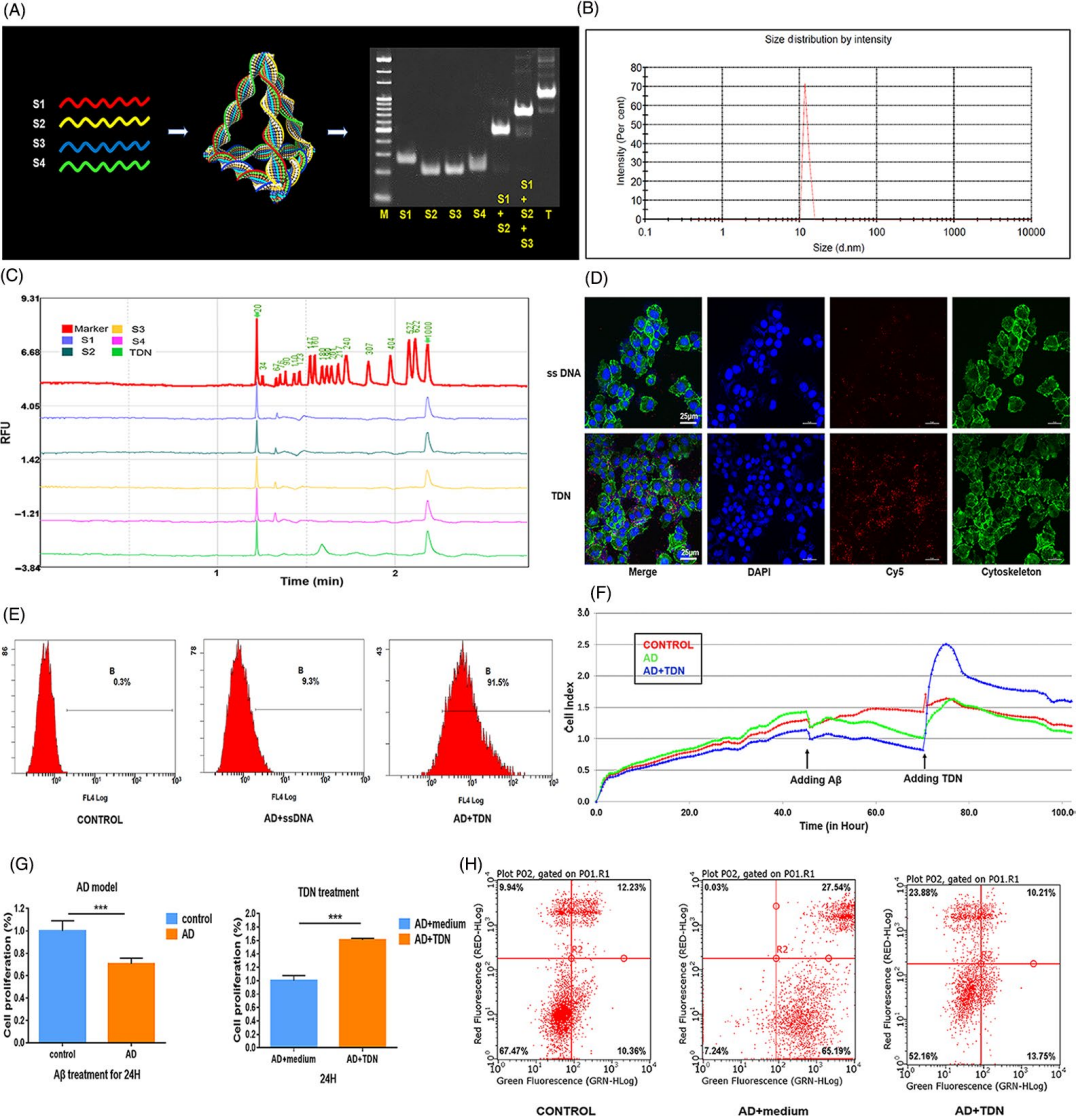
Therapeutic effect of tetrahedral DNA nanostructures (TDN) on an Alzheimer's disease PC12 cell model. A, Sketch map of TDNs and confirmation of the successful synthesis of TDNs by native PAGE (M: marker, S: single‐stranded DNA, T: TDN). B, Confirmation of the successful synthesis of TDNs by capillary electrophoresis. C, Confirmation of the successfully assembled TDNs by dynamic light scattering. D, Cellular uptake of Cy5‐TDNs and Cy5‐single‐stranded DNA in Alzheimer's disease cell model (cytoskeleton: green; nucleus: blue; Cy5: red). Scale bars are 25 µm. E, Cellular uptake of TDNs by flow cytometry. F, Cellular proliferation reaction detected by real‐time cell analysis system (RTCA). Statistical analysis of RTCA results. Data are presented as mean ± standard deviation (n = 4). Student's *t* test: ***P < .001. G, Analysis of apoptosis in an Alzheimer's disease PC12 cell model upon exposure to tetrahedral DNA nanostructures (TDNs; 250 nmol/L) by flow cytometry. H, Addition of TDNs significantly reduced the proportion of apoptotic cells in the Alzheimer's disease cell model, indicating that TDNs can inhibit apoptosis to a certain extent

Figure 1A shows a schematic diagram of TDN synthesis, with each DNA single strand forming one side of the structure. We verified the synthesis of the tetrahedrons by non‐denaturing polypropylene gel electrophoresis. Figure 1B shows the results of capillary electrophoresis detection of four DNA single strands and the molecular weight of TDN. The size of DNA single strands and TDNs is consistent with the results reported in the literature, 50 bp and 200 bp, respectively.^9^, ^14^ TDN particle size was about 12 nm as assessed by DLS (Figure 1C). Based on these tests, we confirmed the successful production of TDNs.

### 3.2 TDN has a therapeutic effect on an in vitro Alzheimer's disease model

According to reports in the literature,^9^ compared to traditional DNA single strands, TDNs enter living cells through caveolin‐mediated endocytosis and maintain their structural integrity for a period of time inside the cell.^9^ We first confirmed that TDNs, rather than DNA single strands, successfully entered PC12 cells, by testing their cellular uptake by confocal microscopy and flow cytometry (Figure 1D,E). Next, we treated PC12 cells with Aβ25‐35 at a concentration of 25 μmol/L for 24 hour to generated an Alzheimer's disease cell model based on previous experiments^16^ and confirmed successful modelling by RTCA. As shown in Figure 1F,G, Aβ25‐35 treatment significantly reduced (to about 68%) cell viability, indicating successful Alzheimer's disease modelling in PC12 cells. To examine whether TDNs had any therapeutic effect on this Alzheimer's disease cell model, we treated cells with either serum‐free medium or with TDNs. After 24 hours of TDNs treatment, the cell viability increased by about 60%, indicating that TDNs exert a significant therapeutic effect.

### 3.3 TDN treatment reduces Alzheimer's disease‐induced apoptosis in vitro

**Figure 2:**
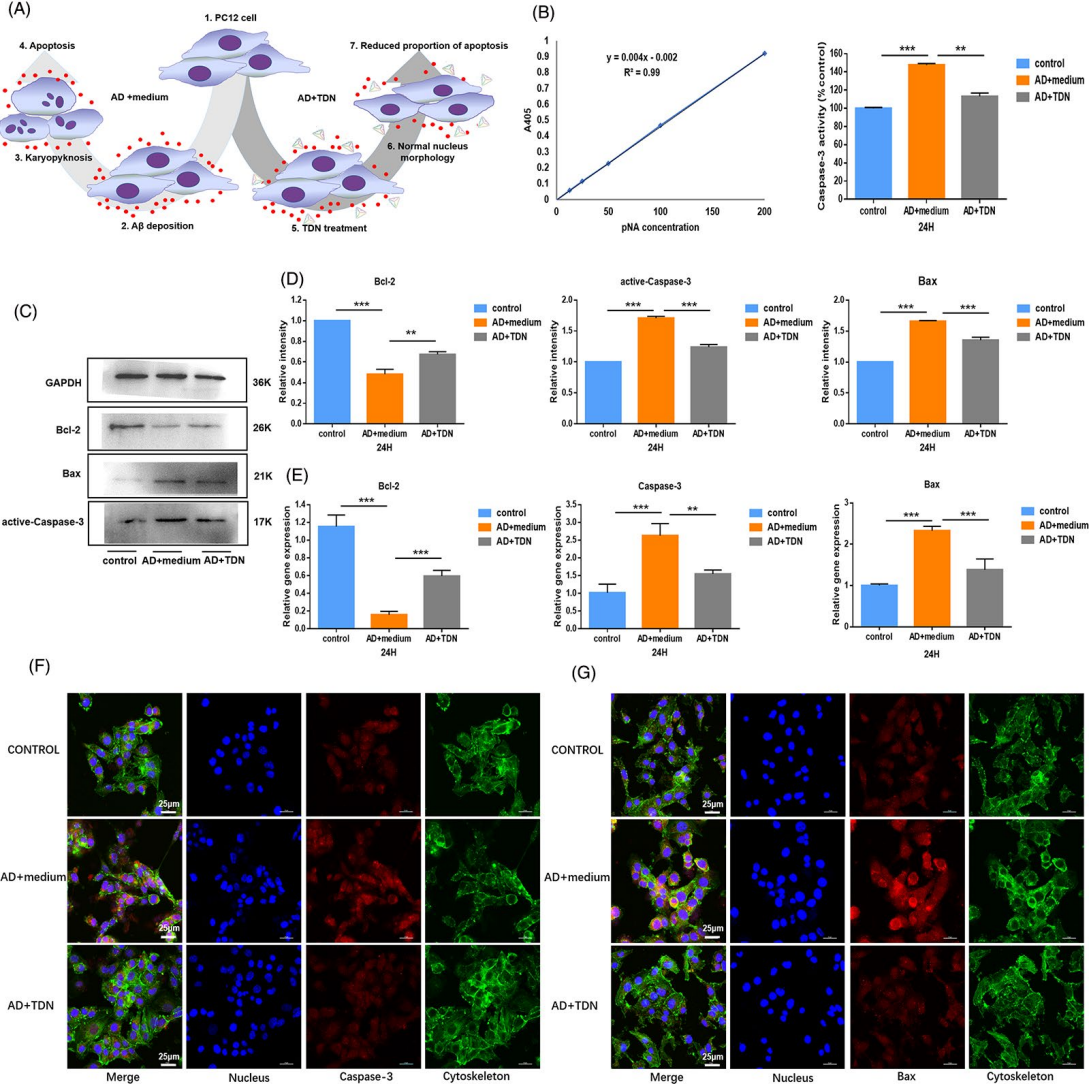
Detection of apoptosis‐related proteins and genes. A, A schematic diagram showing the treatment effect of TDNs in an Alzheimer's disease cell model. 1, 2, 3 and 4 represent the process of establishing Alzheimer's disease cell models. 1, 5, 6 and 7 represent the process of TDNs treated Alzheimer's disease cell models. B, Changes in caspase‐3 activity detected by caspase‐3 Activity Assay Kit. C, Western blot analysis of protein expression levels upon exposure to TDNs (250 nmol/L) for 24 h. D, Quantification of protein expression levels upon exposure to TDNs (250 nM) for 24 h. Data are presented as mean ± SD (n = 4). Student's *t* test: **P < .01, ***P < .001. E, Quantification of gene expression levels upon exposure to TDNs (250 nmol/L) for 24 h. Data are presented as mean ± standard deviation (SD; n = 4). Student's *t* test: **P < .01, ***P < .001. F, Photomicrographs showing treated Alzheimer's disease cell model (Cytoskeleton: green, Nucleus: blue, Caspase‐3: red). Scale bars are 25 µm. G, Photomicrographs showing treated Alzheimer's disease cell model (Cytoskeleton: green, Nucleus: blue, Bax: red). Scale bars are 25 µm

To explore the mechanism of TDNs action, we assessed apoptosis by flow cytometry, as well as the expression of proteins and genes related to the apoptotic signalling pathway by Western blot, caspase‐3 Activity Assay Kit (Beyotime) and real‐time qPCR. Addition of TDNs significantly reduced the proportion of apoptotic cells in the Alzheimer's disease cell model, indicating that TDNs can inhibit apoptosis to a certain extent (Figure 1H). Moreover, the caspase‐3 activity was decreased after TDN addition (Figure 2A,B).

Bax is one of the major apoptotic genes in mitochondria‐dependent apoptosis. It enhances the permeability of mitochondria, releases cytochrome C from mitochondria, mediates the formation of the apoptotic complex and activates caspases 3/6/7.^5^, ^20^ In contrast, Bcl‐2 reduces mitochondrial permeability by forming a dimmer with Bax, thus inhibiting apoptosis.^21^, ^22^, ^23^ Thus, we subsequently examined the expression of Bax, Bcl‐2 and caspase‐3 by Western blot and real‐time qPCR. Figure 2C,D show that the protein expression levels of the apoptosis mediators Bax and caspase‐3 were significantly decreased after TDN treatment. In contrast, the expression of the anti‐apoptotic protein Bcl‐2 was significantly increased. The mRNA analysis yielded consistent results (Figure 2E). To further visualize changes in the expression of caspase‐3 and Bax after TDN addition, we performed an immunofluorescence assay. In agreement with our previous findings, the levels of both proteins were significantly reduced after TDN treatment (Figure 2F,G).

### 3.4 TDNs can cross the BBB

The blood‐brain barrier (BBB) separates the central nervous system (CNS) from the blood circulation and prevents entry of substances in the plasma that are toxic to the brain, thereby maintaining a good living environment for nerve cells. However, the BBB does not prevent all substances from entering the brain tissue. For example, essential nutrients (such as glucose, amino acids.) may enter the CNS faster than with simple diffusion.^24^, ^25^

**Figure 3:**
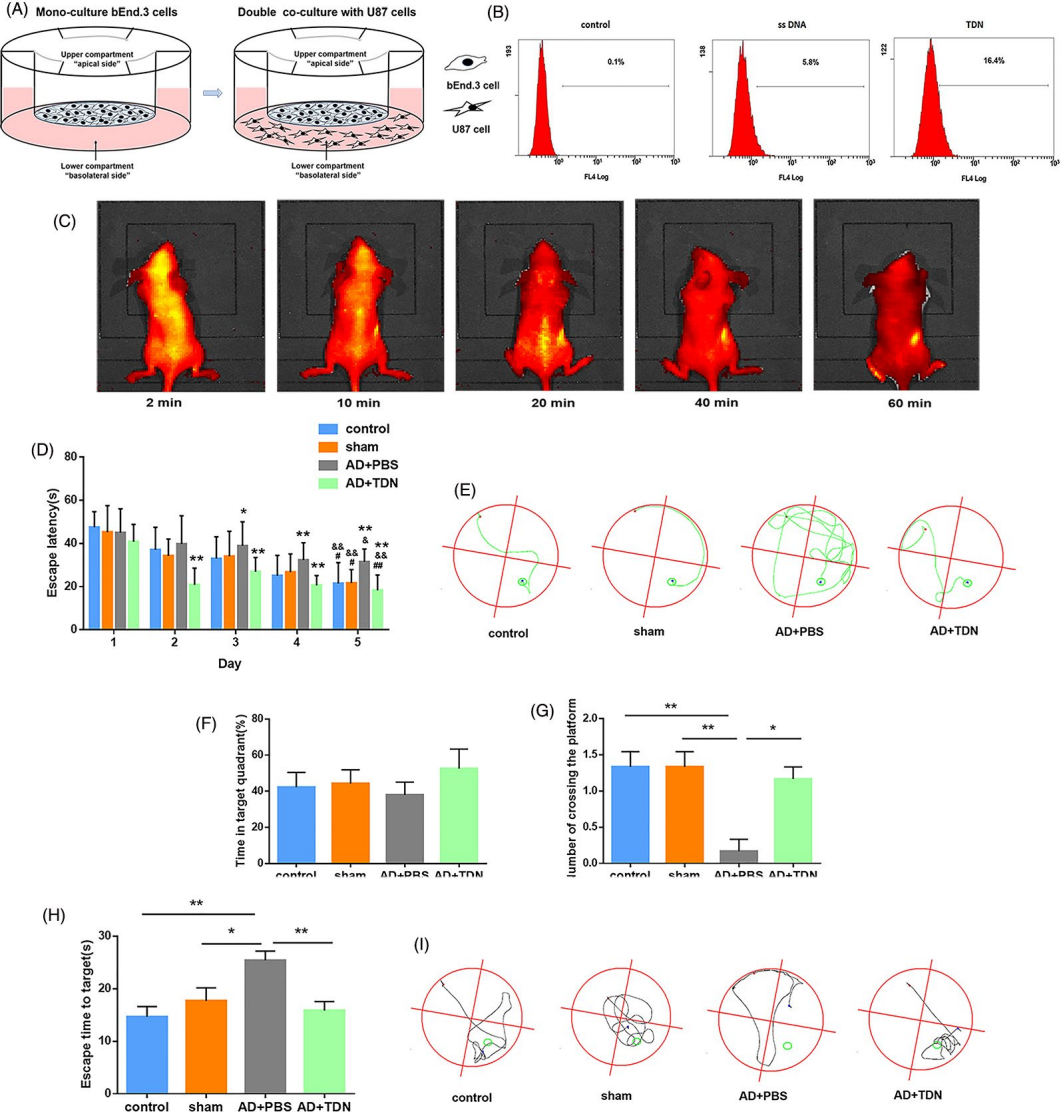
Behavioural test results. A, Schematic diagram of the BBB pattern in vitro. B, Flow cytometry results. C, Detection of the in vivo distribution of TDNs in rats after tail‐vein injection by small animal in vivo imaging. D, Escape latency in the positioning navigation experiment for each group. *P < .05, **P < .01 vs control; ^#^ *P* < .05, ^##^ *P* < .01 vs Alzheimer's disease + PBS Day 5; ^&^ *P* < .05, ^&&^ *P* < .01 Day 5 vs Day 1. E, Trajectory map of rats after entering the water from the second quadrant during the positioning navigation test on the 5th day. F, Percentage of total time spent in the target quadrant during the space exploration experiment in each group. Data are presented as mean ± standard deviation (SD; n = 6). Student's *t* test was used for statistical analysis. G, The number of times the rats crossed the platform in each group in the space exploration experiment. Data are presented as mean ± SD (n = 6). Student's *t* test: *P < .05, **P < .01. H, Escape time to target. Data are presented as mean ± SD (n = 6). Student's *t* test: *P < .05, **P < .01. I, Trajectory map of rats in the space exploration experiment

Before conducting in vivo experiments, we assessed whether TDNs can cross the BBB. First, we constructed an in vitro BBB model by co‐culturing the brain microvascular endothelial cell line Bend.3 with PC 12 cells and tested the ability of TDNs to cross the BBB by flow cytometry. As shown in Figure 3A,B, compared to single‐stranded DNA, TDNs could indeed cross the BBB. Subsequently, we injected the TDN solution into the tail vein of rats, and using small animal in vivo imaging technology, we observed the distribution of TDNs in vivo at different time points. As shown in Figure 3C, TDNs accumulated in the brain in the first 10 minutes after injection and then gradually decreased. The above experimental results confirm that TDNs can partially cross the BBB, providing an experimental basis for the subsequent in vivo experiments.

### 3.5 TDNs improve learning and memory in an Alzheimer's disease rat model

To detect behavioural changes, we subjected rats of each group to the Morris water maze test. Figure 3D,E show the experimental results and trajectories of the directional navigation experiments for the five consecutive days. Compared with the first day, the escape latency of each group was reduced over time; however, there was a significant difference among control, sham‐operated and the TDN‐injected rats on the 5th day (*P* < .01). On the second day, the escape latency of Alzheimer's disease rats was significantly higher (*P* < .05), while that of TDN‐injected rats was significantly lower (*P* < .05) than that of control rats. For the days 3, 4 and 5, this difference was more pronounced. On the fifth day, the escape latency was significantly lower in the control, sham and TDN groups than in the Alzheimer's disease group (*P* < .05). The difference was most significant for the TDN injection group (*P* < .01).

Figure 3F‐I shows the results of the space exploration experiment and the trajectory on the 6th day. The time spent in the target quadrant was higher in rats of the control, sham and TDN groups than those in the Alzheimer's disease group, although the difference between TDN‐injected and Alzheimer's disease rats was not statistically significant (*P* > .05; Figure 3F). Moreover, the number of crossings was greater in these groups than in the Alzheimer's disease group, with a statistically significant difference found between the TDN and Alzheimer's disease groups (*P* < .05; Figure 3G). Lastly, the latency to find the platform was lower in all groups compared to the Alzheimer's disease group, which was statistically significant between the TDN and Alzheimer's disease groups (*P* < .05; Figure 3H). Taken together, the above results suggest that TDNs can improve the learning and memory ability of Alzheimer's disease rats, which will provide new research ideas for the treatment of Alzheimer's disease.

### 3.6 TDNs partially restore Alzheimer's disease‐related morphological anomalies in the hippocampus

**Figure 4:**
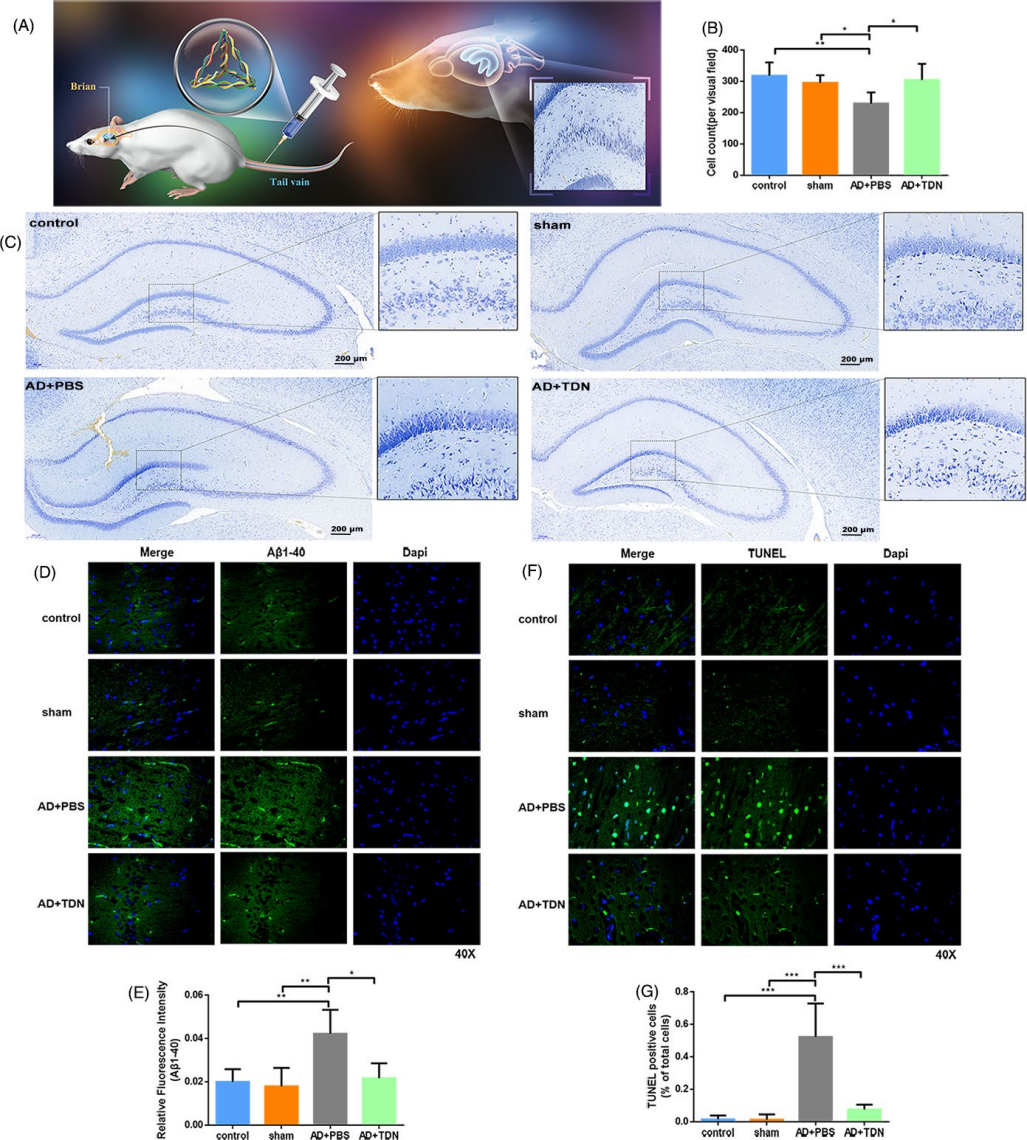
Examination of amyloid β deposition and apoptosis after tetrahedral DNA nanostructures (TDN) treatment. A, Schematic diagram of tail‐vein administration. B, Representative Nissl‐stained sections of hippocampus for each group of rats. Dark blue particles: Nissl body, light blue: nucleus, background: substantially colourless. C, Statistical analysis of the number of neurons in the hippocampus of each group of rats. D, Immunofluorescence staining for Aβ1‐40 in the hippocampus of each group of rats. E, Statistical analysis of the number of Aβ1‐40‐positive plaques in the hippocampus of each group. Data are presented as mean ± standard deviation (n = 6). Student's *t* test: *P < .05, **P < .01. F, TUNEL staining in the hippocampus for each group. G, Statistical analysis of TUNEL‐positive cells in the hippocampus. Data are presented as mean ± standard deviation (SD; n = 6). Student's *t* test: *P < .05, **P < .01, ***P < .001

In order to detect changes in the morphology and number of neurons in the hippocampus after TDN injection in the tail vein, we performed Nissl staining. Figure 4 shows representative Nissl‐stained hippocampal sections (Figure 4A,C) and the statistical analysis for the number of neurons in the hippocampus (Figure 4B) for each group of rats. The number of neurons was significantly higher in the control, sham and TDN groups than in the Alzheimer's disease group (*P* < .05) for the same area examined; in addition, the morphology of the Nissl‐stained cell bodies was abnormal in the Alzheimer's disease group. Both the number of neurons and the number of abnormal cell bodies changed after TDN treatment, indicating that TDN tail‐vein injection restores, in some degree, the anomalies in cell number and morphology caused by Alzheimer's disease.

### 3.7 TDNs reduce Aβ_1‐40_ deposition in the rat hippocampus

Current research shows that Aβ aggregation into senile plaques, abnormal accumulation of the intracellular protein Tau into neurofibrillary tangles (NFT), and neuronal death represent the main pathological features in Alzheimer's disease patients.^26^ The accumulation of Aβ in the brain is the main cause of induction and aggravation of Alzheimer's disease, which ultimately induces cytotoxicity and cell death, including mitochondria‐dependent apoptosis.^27^, ^28^ Therefore, inhibiting the formation, deposition and toxic effects of Aβ may be the fundamental strategy for the treatment of Alzheimer's disease. We thus examined the expression of Aβ_1‐40_ protein in the rat hippocampus and measured the number of Aβ_1‐40_‐positive plaques for each group (Figure 4D,E). Aβ_1‐40_ expression was higher in the Alzheimer's disease than in the control and sham groups. However, TDN injection into the tail vein significantly decreased Aβ_1‐40_ expression in the Alzheimer's disease group.

### 3.8 TDN treatment inhibits Alzheimer's disease‐induced apoptosis in the hippocampus

Next, in order to further explore the effect of TDNs on cell apoptosis in vivo, we performed TUNEL staining of the rat hippocampus. Figure 4F,G show representative TUNEL‐stained sections of the hippocampus and the statistical analysis of positive cells for each group. There was no significant difference in apoptosis levels in the hippocampus between the sham and the control groups. However, significantly higher apoptosis levels were found in the Alzheimer's disease and TDN‐treated groups and the level of apoptosis in the Alzheimer's disease model group was significantly higher than that in the TDN treatment group. The above results indicated that TDN tail‐vein injection inhibits Alzheimer's disease‐induced apoptosis of hippocampal cells.

**Figure 5:**
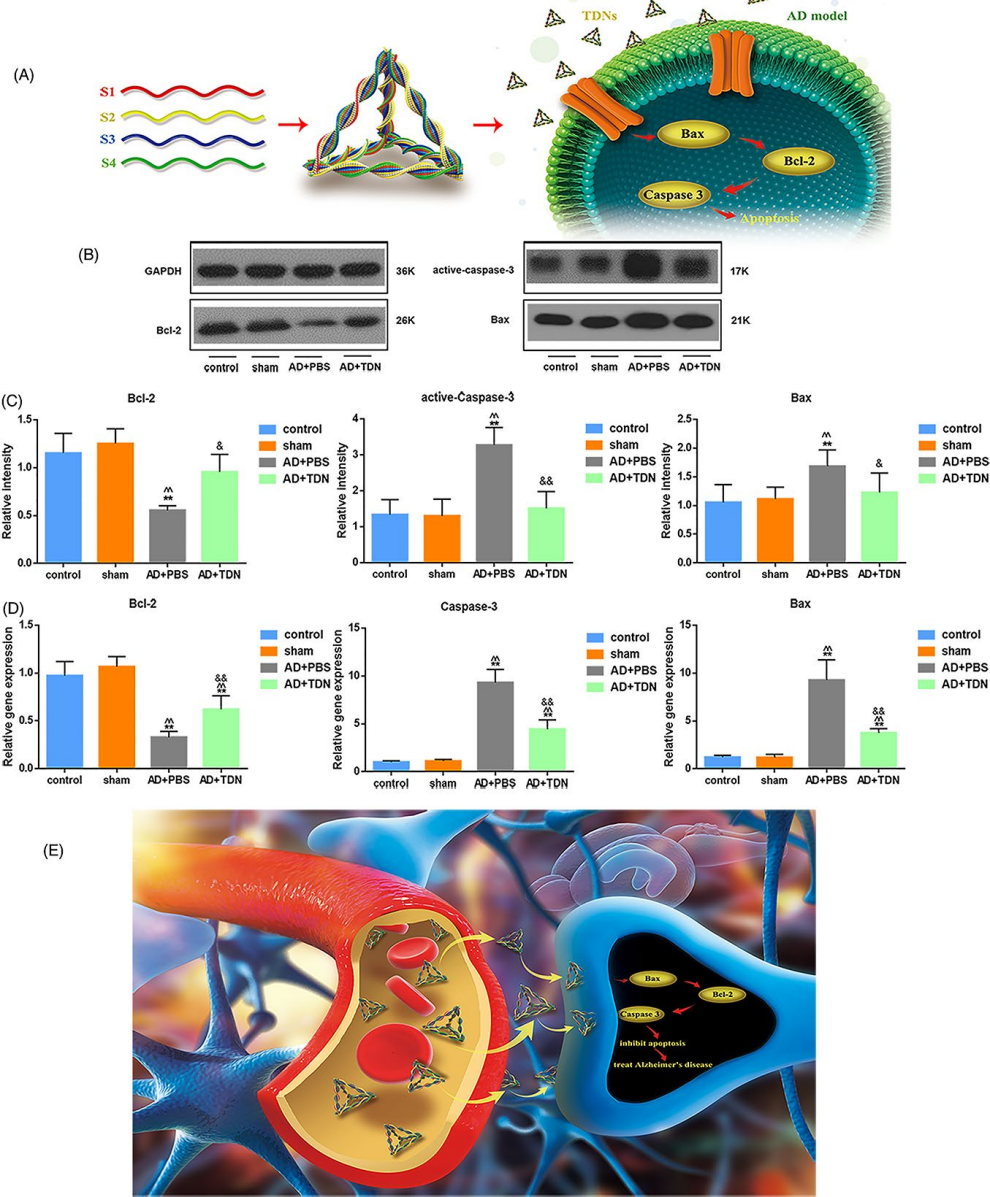
Examination of apoptosis‐related signalling pathway after tetrahedral DNA nanostructures (TDN) treatment. A, Schematic diagram of the effect of TDN on apoptotic signalling pathway in Alzheimer's disease model. B, Western blot analysis of the protein expression levels of active caspase‐3, Bcl‐2 and Bax in the hippocampus for each group. C, Quantification of protein expression levels in the hippocampus. Data are presented as mean ± SD (n = 6). Student's *t* test: *P < .05, **P < .01 vs control; ^^^ *P* < .05, ^^^^ *P* < .01 vs sham; ^&^ *P* < .05, ^&&^ *P* < .01 vs Alzheimer's disease + PBS. D, Quantification of gene expression in the hippocampus. Data are presented as mean ± SD (n = 6). Student's *t* test: *P < .05, **P < .01 vs control; ^^^ *P* < .05, ^^^^ *P* < .01 vs sham; ^&^ *P* < .05, ^&&^ *P* < .01 vs Alzheimer's disease + PBS. E, Schematic diagram of the therapeutic effect of TDN on Alzheimer's disease model

Next, we used Western blot and real‐time qPCR analysis to assess the expression levels of active caspase‐3, Bcl‐2 and Bax in the rat hippocampus (Figure 5A‐D). No difference was found between the control and sham group (*P* > .05). In contrast, both the protein and mRNA expression levels of active caspase‐3 and Bax were significantly higher in the Alzheimer's disease than in the control group and sham groups, while Bcl‐2 expression levels were significantly lower (*P* < .01). TDN injection in Alzheimer's disease rats significantly reversed these changes, restoring the expression of active caspase‐3, Bax and Bcl‐2 to the levels of the control and sham groups (*P* < .01).

## 4 DISCUSSION

Drugs that are currently used for Alzheimer's disease are all symptomatic treatment drugs that do not promote nerve cell proliferation or inhibit their apoptosis.^29^, ^30^ Existing drugs may have certain toxic side effects on cells, and thus, their biocompatibility, biosafety and biodegradability are poor. Moreover, neural stem cells are characterized by low survival rate, slow proliferation, and slow differentiation and maturity.^31^, ^32^ Promoting nerve regeneration through small molecule compounds may be a new therapeutic strategy.

Nucleic acid nanotechnology is a hot topic in recent years.^33^, ^34^ Due to the special molecular properties of DNA, it has great potential for development in many research directions such as biomedicine. They generally have low cytotoxicity and strong resistance to enzymatic degradation in biological environments, which facilitate the in vivo application of DNA nanostructures.

Our in vitro and in vivo experiments confirmed that, in a specific concentration range, TDNs have no toxic and side effects on nerve cells, can effectively promote cell survival, inhibit apoptosis and reduce deposition of Aβ. TDNs can inhibit the level of reactive oxygen species in the Alzheimer's disease model, thereby inhibiting the expression of Bax, attenuating the activation of Caspase, inhibiting the apoptosis‐related signalling pathway and finally inhibiting apoptosis. Moreover, TDNs partially passed the blood‐brain barrier and improved memory and learning in our Alzheimer's disease rat model. Our findings suggest that TDNs may be used as a new drug for the treatment of Alzheimer's disease (Figure 5E). This novel and noteworthy discovery may pave the way for the potential use of TDNs in preventing neuronal cells death in the process of Alzheimer's disease.

## CONFLICT OF INTEREST

The authors declare no competing interests.

## AUTHOR CONTRIBUTION

All authors contributed to the study concept and design. Xiaoru Shao and Weitong Cui carried out the in vivo experiments on Alzheimer's disease rat model. Xiaoru Shao, Xueping Xie and Yuxi Zhan carried out the in vitro experiments on Alzheimer's disease cell model. Wenjuan Ma and Yuxi Zhan collected the data. Xiaoru Shao performed the analysis and drafted the manuscript. Yunfeng Lin initiated the research, designed research studies, analysed data and provide the funding. All authors have reviewed and approved the manuscript.

## Data Availability

The data that support the findings of this study are available from the corresponding author upon reasonable request.
